# Femoral geometry, bone mineral density, and the risk of hip fracture in premenopausal women: a case control study

**DOI:** 10.1186/s12891-016-0893-2

**Published:** 2016-01-25

**Authors:** Dong-Hwa Lee, Kyong Yeun Jung, A Ram Hong, Jung Hee Kim, Kyoung Min Kim, Chan Soo Shin, Seong Yeon Kim, Sang Wan Kim

**Affiliations:** Department of Internal Medicine, Seoul National University Hospital, 101 Daehak-ro, Jongno-gu, Seoul, 110-744 Korea; Department of Internal Medicine, Seoul National University Bundang Hospital, 300 Gumi-dong, Bundang-gu, Seongnam-si, Kyunggi-do 463-707 Korea; Department of Internal Medicine, Seoul Metropolitan Government Boramae Medical Center, 20 Boramae-Ro 5-Gil, Dongjak-Gu, Seoul, 156-707 Korea

**Keywords:** Hip geometry, Hip fracture, Hip axis length, Premenopausal women

## Abstract

**Background:**

The purpose of this study was to determine the relationships among hip geometry, bone mineral density, and the risk of hip fracture in premenopausal women.

**Methods:**

The participants in this case–control study were 16 premenopausal women with minimal-trauma hip fractures (fracture group) and 80 age-and BMI-adjusted controls. Subjects underwent dual-energy X-ray absorptiometry (DXA) to assess BMD at the proximal femur and to obtain DXA-derived hip geometry measurements.

**Results:**

The fracture group had a lower mean femoral neck and total hip BMD than the control group (0.721 ± 0.123 vs. 0.899 ± 0.115, *p* <0.001 for the femoral neck BMD and 0.724 ± 0.120 vs. 0.923 ± 0.116, *p* <0.001 for the total hip BMD). In addition, participants in the fracture group had a longer hip axis length (HAL; *p* = 0.007), narrower neck shaft angle (NSA; *p* = 0.008), smaller cross sectional area (CSA; *p* < 0.001) and higher cross sectional moment of inertia (CSMI; *p* = 0.004) than those in control group. After adjusting for BMD, the fracture group still had a significantly longer mean HAL (*p* = 0.020) and narrower NSA (*p* = 0.006) than the control group.

**Conclusions:**

BMD is an important predictor of hip fracture in premenopausal women. Furthermore, HAL and NSA are BMD-independent predictors of hip fracture in premenopausal women. Hip geometry may be clinically useful for identification of premenopausal women for whom active fracture prevention should be considered.

## Background

Premenopausal women with low bone mineral density (BMD) are increasingly being identified [1]. Although low BMD is a major risk factor for fracture in postmenopausal women, the clinical significance of low BMD prior to menopause is not known. Low hip BMD has been shown to predict hip fracture in postmenopausal and perimenopausal women [2–5], however, the relationship between BMD and fracture in premenopausal women is unclear. Currently, a diagnosis of osteoporosis in premenopausal women is made based only on clinical signs, and not BMD [6].

Fractures are caused by forces that exceed bone strength, and can occur at any age. Bone strength is determined by bone geometry, microarchitecture, and material properties such as BMD. Hip fracture is one of the most serious and undesirable outcomes of low bone strength [7]. Dual energy X-ray absorptiometry (DXA) scans can provide hip geometric characteristics as well as classical BMD measurements [8, 9].

Hip structural analysis (HSA) is used to extract cross-sectional geometry data from DXA images of the proximal femur to calculate bone strength [10, 11]. Previous reports have suggested that there are associations between hip geometry and hip fracture risk in postmenopausal women [12–15]. Moreover, hip axis length (HAL), the distance from the base of greater trochanter to the inner pelvic rim, has been shown to be strongly correlated with the risk of hip fracture [16–20]. Whether HSA could be useful for clinical assessment of bone fragility prior to menopause remains unclear. Because hip fracture, although serious, is very uncommon in premenopausal women, little is known about the relationship between hip geometry and minimally traumatic hip fracture risk in premenopausal women. Thus, the purpose of this study was to determine the relationship between hip geometry, BMD, and the risk of hip fracture in premenopausal women.

## Methods

### Subjects

**Fig. 1:**
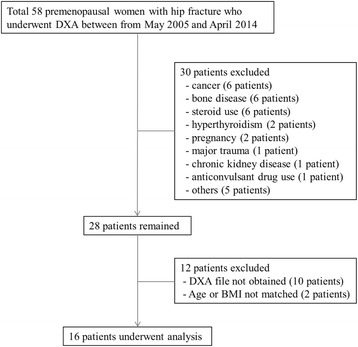
Flow chart describing process of patient enrollment

A total of 16 female patients with hip fracture (fracture group) were enrolled for participation in this study through retrospective review of medical records from May 2005 to April 2014 at Seoul National University Hospital, Seoul National University Bundang Hospital, and Seoul Metropolitan Government Boramae Medical Center. Figure 1 illustrates process of the patient enrollment. Only patients with fractures due to minor trauma, such as falls from a standing position or while walking, were included. Thirteen patients had femoral neck fractures, two patients had intertrochanteric fractures, and one patient had distal femur fracture. The control group consisted of 80 age- and body mass index (BMI)-matched female subjects from the Healthcare System Gangnam Center. BMD and geometrical parameters were also obtained from 2005 to 2014 in the control group. Premenopausal controls have undergone BMD for health check-up, although the Korean Society of Bone and Mineral Research guidelines do not suggest BMD should be routinely screened in young women. Menopausal status was self-reported or, in cases in which this information was missing, we assumed that women younger than 45 years were premenopausal. Exclusion criteria included the presence of secondary causes of osteoporosis (such as hyperthyroidism, Cushing’s syndrome, rheumatoid arthritis, systemic lupus erythematosus, chronic kidney disease), a history of malignant disease, or medications (such as steroids or anticonvulsants) known to alter calcium and bone metabolism. The study was approved by the ethics committees of Seoul National University Hospital, Seoul National University Bundang Hospital, and Seoul Metropolitan Government Boramae Medical Center.

### Measurements

Bone mineral density, hip geometry, and demographic data, including age, height, and body weight were collected. Measurements of BMD were made by DXA (Prodigy™, Lunar Corporation, Madison, WI, USA) at the proximal femur in all subjects capable of lying in a supine position with their hips extended and internally rotated 15°. In the fracture group, the mean time between the DXA scan and the fracture was 65 days (range, 0–533 days). Hip geometry measurements at the specific region of interest, including narrow neck, intertrochanteric, and femoral shaft measurements, were made using the standard DXA hip image with Encore software version 11.0, as previously described [11]. The measured parameters were: HAL, the distance from the pelvic rim to the outer margin of the greater trochanter along the axis of the femoral neck; neck shaft angle (NSA), the angle between the derived axes of the femoral neck and shaft; cross sectional area (CSA), the amount of (cortical equivalent) bone surface area in the cross-section after excluding all trabecular and soft tissue spaces; cross sectional moment of inertia (CSMI), an index of structural rigidity that reflects distribution of mass about the center of a structural element; buckling ratio (BR), the relative thickness of the cortex as an estimate of cortical stability in buckling; and section modulus (SM), an indicator of the ability of the bone to resist maximum bending stress in the image plane.

### Statistical analysis

All continuous data are presented as mean ± standard deviation. The significance of differences between the fracture and control groups was assessed using the Mann–Whitney *U* test. Analysis of covariance with correction for BMD was then performed. The relationships among bone geometric properties were evaluated using Pearson’s correlation coefficients. Univariate logistic regression analyses were used to evaluate the influence of BMD and geometry parameters on fracture risk. Statistical analyses were performed using SPSS version 22.0 software and differences were considered significant if *p* <0.05.

## Results

### Subject characteristics

The baseline characteristics of the study populations are presented in Tables 1 and 2. Age, height, weight, and BMI did not significantly differ between groups. The mean age of the patients with hip fractures was 35.4 ± 6.0 years and their mean BMI was 19.2 ± 2.7 kg/m^2^. Compared with their age- and BMI-matched controls, the patients in the fracture group had a significantly lower mean femoral neck BMD (0.721 ± 0.123 vs. 0.899 ± 0.115, *p* < 0.001) and total hip BMD (0.724 ± 0.120 vs. 0.923 ± 0.116, *p* < 0.001), longer HAL (105.72 ± 4.88 vs. 101.80 ± 4.91, *p* = 0.007), narrower NSA (123.09 ± 2.81 vs. 126.07 ± 4.56, *p* = 0.008), smaller CSA (105.59 ± 15.85 vs. 130.92 ± 17.99, *p* < 0.001), and lower CSMI (7031.12 ± 1630.76 vs. 8521.96 ± 1652.57, *p* = 0.004). After adjusting for femoral neck BMD, the HAL and NSA were still significantly different between groups (*p* = 0.020 for HAL and *p* = 0.006 for NSA).

**Table 1:** Baseline characteristics of the study population

	Fracture group	Control group	*P* value
N	16	80	
Age (year)	35.4 ± 6.0	35.4 ± 5.8	0.992
Height (cm)	163.96 ± 0.04	161.97 ± 0.05	0.288
Weight (kg)	51.0 ± 7.7	51.2 ± 5.4	0.871
BMI (kg/m^2^)	19.2 ± 2.7	19.6 ± 2.2	0.609
Calcium (mg/dL)	8.8 ± 0.5	9.1 ± 0.4	0.049
Phosphorus (mg/dL)	3.6 ± 0.5	3.5 ± 0.4	0.898
25-Hydroxyvitamin D (ng/mL)	17.5 ± 9.8	22.2 ± 6.8	0.228
BUN (mg/dL)	12.0 ± 5.8	12.1 ± 3.2	0.560
Cr (mg/dL)	0.69 ± 0.12	0.79 ± 0.10	0.001
WBC (10^3^/μL)	6.43 ± 1.96	5.06 ± 1.57	0.006
Hemoglobin (g/dL)	11.6 ± 1.7	12.7 ± 1.1	0.009
Platelet (10^3^/μL)	250.0 ± 48.1	254.7 ± 52.4	0.836

Data are means ± SD

*P* value for Mann–Whitney *U* test

**Table 2:** Bone mineral density and geometry measurements

	Fracture group	Control group	*P* value	^a^ *P* value
Femoral neck BMD (g/cm^2^)	0.721 ± 0.123	0.899 ± 0.115	<0.001	
Total hip BMD (g/cm^2^)	0.724 ± 0.120	0.923 ± 0.116	<0.001	
Hip axis length (mm)	105.72 ± 4.88	101.80 ± 4.91	0.007	0.020
Neck shaft angle (degree)	123.09 ± 2.81	126.07 ± 4.56	0.008	0.006
Cross sectional area (mm^2^)	105.59 ± 15.85	130.92 ± 17.99	<0.001	0.710
Cross sectional moment of inertia (mm^4^)	7031.12 ± 1630.76	8521.96 ± 1652.57	0.004	0.322
Minimal neck width (mm)	28.70 ± 2.10	28.78 ± 1.60	0.743	0.486
Cortical thickness of femoral neck (mm)	3.77 ± 1.38	4.33 ± 1.32	0.141	0.853
Cortical thickness of femoral shaft (mm)	4.05 ± 1.25	4.36 ± 1.06	0.321	0.679
Section modulus (mm^3^)	434.96 ± 89.34	540.46 ± 82.75	<0.001	0.315
Buckling ratio	4.84 ± 1.75	3.96 ± 1.30	0.105	0.849

Data are means ± SD

*P* value for Mann–Whitney *U* test

^a^ *P* value for ANCOVA (adjusted for femoral neck BMD)

### Relationships among hip geometric parameters and BMD

The results of Pearson’s correlation testing of the relationships among geometric parameters and between BMD and geometric parameters are presented in Table 3. Femoral neck BMD was positively correlated with total hip BMD, CSA, CSMI, and SM, and was negatively correlated with BR (*p* < 0.001 for all). There was a positive correlation between HAL and minimal neck width (*p* < 0.001), and CSA was also positively correlated with CSMI and SM (*p* < 0.001 for both). There were no meaningful correlations between NSA and any other parameters.

**Table 3:** Pearson correlations among hip geometric parameters and BMD

	TH BMD	HAL	NSA	CSA	CSMI	MNW	FN CT	FS CT	SM	BR
	(*P* value)	(*P* value)	(*P* value)	(*P* value)	(*P* value)	(*P* value)	(*P* value)	(*P* value)	(*P* value)	(*P* value)
FN BMD	0.955 (<0.001)	−0.171 (0.098)	0.017 (0.867)	0.922 (<0.001)	0.488 (<0.001)	−0.087 (0.399)	0.340 (0.001)	0.282 (0.006)	0.732 (<0.001)	−0.432 (<0.001)
TH BMD	-	−0.180 (0.081)	0.036 (0.730)	0.891 (<0.001)	0.472 (<0.001)	−0.084 (0.416)	0.346 (0.001)	0.348 (0.001)	0.705 (<0.001)	−0.433 (<0.001)
HAL	-	-	−0.149 (0.149)	−0.004 (0.973)	0.231 (0.024)	0.410 (<0.001)	−0.291 (0.004)	0.106 (0.308)	0.134 (0.196)	0.331 (0.001)
NSA	-	-	-	−0.052 (0.618)	−0.028 (0.786)	−0.063 (0.544)	−0.088 (0.396)	−0.103 (0.323)	−0.067 (0.519)	0.123 (0.233)
CSA	-	-	-	-	0.727 (<0.001)	0.240 (0.019)	0.306 (0.003)	0.373 (<0.001)	0.876 (<0.001)	−0.325 (0.001)
CSMI	-	-	-	-	-	0.746 (<0.001)	0.032 (0.761)	0.379 (<0.001)	0.904 (<0.001)	0.095 (0.360)
MNW	-	-	-	-	-	-	−0.118 (0.253)	0.204 (0.047)	0.473 (<0.001)	0.323 (0.001)
FN CT	-	-	-	-	-	-	-	0.025 (0.809)	0.161 (0.120)	−0.856 (<0.001)
FS CT	-	-	-	-	-	-	-	-	0.379 (<0.001)	−0.039 (0.705)
SM	-	-	-	-	-	-	-	-	-	−0.149 (0.149)

*FN BMD*, femoral neck bone mineral density, *TH BMD* total hip bone mineral density, *HAL* hip axis length, *NSA* neck shaft angle, *CSA* cross sectional area, *CSMI* cross sectional moment of inertia, *MNW* minimal neck width, *FN CT* cortical thickness of femoral neck, *FS CT* cortical thickness of femoral shaft, *SM* section modulus

### Hip geometric parameters, BMD, and the likelihood of hip fracture

Table 4 shows the contribution of BMD and bone geometry parameters to fracture risk. Univariate analysis of each variable was used to determine the odds ratio. Fracture risk increased 6.561-fold (*p* < 0.001) and 6.495-fold (*p* < 0.001) with decreases of 1 SD in femoral neck BMD and total hip BMD, respectively. Fracture risk also increased 4.038-fold (*p* < 0.001) and 3.431-fold (*p* = 0.001) with decreases of 1 SD in CSA and SM, respectively. Increases in HAL and NSA increased the risk of hip fracture (OR 2.212, *p* = 0.013 for HAL and OR = 2.377, *p* = 0.017 for NSA).

**Table 4:** Odds ratios for variables associated with hip fracture

	OR	95 % CI of OR		*P* value
		Lower	Upper	
Age^a^	1.000	0.584	1.712	1.000
BMI^b^	1.251	0.690	2.268	0.460
FN BMD^b^	6.561	2.590	16.617	<0.001
TH BMD^b^	6.495	2.688	15.696	<0.001
HAL^a^	2.212	1.182	4.141	0.013
NSA^b^	2.377	1.171	4.826	0.017
CSA^b^	4.038	1.863	8.752	<0.001
CSMI^b^	2.724	1.301	5.706	0.008
Neck width^b^	1.099	0.614	1.967	0.751
FN cortical thickness^b^	1.587	0.812	3.104	0.177
FS cortical thickness^b^	1.236	0.603	2.533	0.563
SM^b^	3.431	1.617	7.280	0.001
BR^a^	1.833	1.012	3.321	0.045

^a^Fracture risk associated with a 1 SD increase in the variable

^b^Fracture risk associated with a 1 SD decrease in the variable

*BMI* body mass index, *FN BMD* femoral neck bone mineral density, *TH BMD* total hip bone mineral density, *HAL* hip axis length, *NSA* neck shaft angle, *CSA* cross sectional area, *CSMI* cross sectional moment of inertia, *SM* section modulus, *BR* buckling ratio

## Discussion

This case–control study of Korean premenopausal women showed that hip geometry is significantly associated with hip fracture, even prior to menopause. The relationship between hip BMD and hip fracture in premenopausal women is unclear. In this study, low hip BMD was still a major risk factor for hip fracture even in premenopausal women. When adjusted for BMD, HAL remained a significant predictor of hip fracture in premenopausal women. To the best of our knowledge, this is the first study to examine the association between HAL and risk of hip fracture in premenopausal women. When multiple logistic regression analysis was performed, only BMD was remained statistically significant (data not shown). However, when multiple logistic regression analysis using only geometric parameters was performed, long HAL and narrow NSA increased fracture risk with statistical significance (OR 0.247 and 4.484 with a 1 SD decrease in the variable, respectively; data not shown). These results showed that BMD is an important factor for fracture risk and geometric parameters, especially HAL and NSA, are additional risk factors to contribute increasing fracture risk.

It is unclear why the young patients in this study suffered fracture due to minor trauma. The patients with fractures showed significantly low bone mass compared to controls. We postulate that patients with fractures may achieve lower peak bone mass. However, why they have a lower peak bone mass is also unknown. As well, altered hip geometry parameters seem to increase susceptibility to hip fracture.

HAL was one of the first geometric measures proposed as a predictor of hip fracture risk in postmenopausal women, independent of BMD at the femoral neck [14]. A previous study found that an increase in HAL equivalent to 1 SD resulted in a 1.9-fold increase in the risk of femoral neck fracture and a 1.6-fold increase in the risk of trochanteric fracture in elderly women [16], this result is similar to our findings. HAL has also been proposed as a possible explanation for the ethnic difference in hip fracture incidence [21]. A recent study confirmed that HAL is a strong predictor of hip fracture in women older than 40 years when adjusted for BMD and FRAX score [22]. The results of the current study suggest that HAL is also a BMD-independent risk factor for hip fracture in premenopausal women. However, although HAL appears to be a BMD-independent predictor of hip fracture in both postmenopausal and premenopausal women, it may not be associated with hip fracture in men. In contrast to postmenopausal women, there is little evidence that men with hip fractures have longer HALs than age-matched controls [14, 23]. The mechanism by which an increase in HAL increases hip fracture risk in women is unclear; the increased risk has been shown to persist after adjustment for height and weight [16]. Moreover, men generally have a longer HAL than women. Collectively, these findings suggest that HAL is a body size-independent risk factor. It has been proposed that HAL is indicative of the ability of the femur or pelvis to absorb the impact of a fall [24]. The greater trochanter of the femur may extend further beyond the pelvis in people with a longer HAL than in those with a shorter HAL, causing hips with a longer HAL to be predisposed to non-traumatic fracture. Thus, HAL may be of clinical value in initial fracture risk assessment for premenopausal women.

In this study, the NSA was decreased in the fracture group compared with the control group; however, the reason for this is unclear. In a case–control study, NSA was significantly greater in postmenopausal women with femoral neck fractures than in women with trochanteric fractures [25]. However, in this study, 13 of 16 subjects had femoral neck fractures. Previous studies of postmenopausal women have yielded conflicting results regarding the relationship between NSA and hip fracture [16, 25–28]. A recent study suggested that acute NSAs are associated with development of atypical femur fractures [29]. Varus hips seem to be susceptible to fracture due to increased lateral cortical loading, and we speculate that this may be a potential mechanism of hip fracture development in the premenopausal women in our study.

In this study, we assumed that women younger than 45 years were premenopausal. The mean age at natural menopause was 50.2 ± 3.7 years from the Korea National Health and Nutrition Examination Survey (KNHANES) [30]. Furthermore, in data from KNHANES 2008–2010, BMD was significantly decreased in women with age over 45 years [31]. In terms of bone mass, we considered that women with age below 45 years were safely premenopause.

This study had several limitations. The most of them result from its small sample size and retrospective design. As well, we selected only age-and BMI-matched controls, clinical information of subjects were insufficient and confounding factors were not strictly controlled. However, the fact that the incidence of hip fracture prior to menopause is extremely rare should be considered.

## Conclusions

The results of this study suggest that BMD is an important risk factor for hip fracture in premenopausal women. Furthermore, long HALs and acute NSAs are BMD-independent risk factors for hip fracture in premenopausal women. Hip geometry may be clinically useful for identification of premenopausal women for whom active fracture prevention should be considered.

## Abbreviations

BMD: bone mineral density
BMI: body mass index
BR: buckling ratio
CSA: cross sectional area
CSMI: cross sectional moment of inertia
DXA: Dual energy X-ray absorptiometry
HAL: hip axis length
HSA: Hip structural analysis
NSA: neck shaft angle
SM: section modulus
